# Female exposed to the Chinese famine increases the risk of dyslipidemia in later life

**DOI:** 10.1097/MD.0000000000034262

**Published:** 2023-07-07

**Authors:** Huali Xiong, Daiqiang Liu, Dayi Tang, Fengxun Ma

**Affiliations:** a Department of Non-Communicable Chronic Disease Prevention and Control, Rongchang Center for Disease Control and Prevention, Rongchang, Chongqing, China; b Department of Hospital Information, The People’s Hospital of Rongchang District, Rongchang, Chongqing, China; c First Clinical College, Mudanjiang Medical College, Mudanjiang, Heilongjiang, China.

**Keywords:** Chinese famine, dyslipidemia, gender difference, undernutrition

## Abstract

The Developmental Origins of Health and Disease theory suggests that early-life malnutrition is associated with an increased risk of chronic disease in adulthood. In this study, we aimed to analyze the association between exposure to the Chinese famine during fetal, childhood, and adolescence, while also exploring potential gender disparities in this association.

From August 2018 to 2022 December, a 3-stage stratified random sampling method was employed to recruit 6916 eligible participants in Chongqing for this study. The participants were enrolled into 4 cohorts based on their birthdates: non-exposed, fetal-exposed, childhood-exposed, and adolescence-exposed. Participants were defined as having dyslipidemia according to the 2016 Chinese guideline for the management of dyslipidemia in adults, as well as self-reported dyslipidemia. In total, 6916 eligible participants were interviewed, including 1686 participants exposed when fetal, 1626 participants exposed during childhood, 1648 participants exposed during adolescence, and 1956 participants who had no exposure.

The prevalence of dyslipidemia in the non-exposed, fetal-exposed, childhood-exposed, and adolescence-exposed cohorts was 21.43%, 25.00%, 24.38%, 22.52% in males and 20.00%, 36.57%, 34.60%, 32.59% in females, respectively. There was an increased risk of dyslipidemia among females exposed to the Chinese famine during the fetal (odds ratio [OR] = 1.613, 95% confidence interval [CI]: 1.179–2.205), childhood (OR = 1.857, 95% CI: 1.384–2.491), adolescence (OR = 1.531, 95% CI: 1.137–2.060) stage, However, no significant association was observed in male adults.

Exposure to the Chinese famine during fetal, childhood, and adolescence stages increases the risk of dyslipidemia in adulthood in females, but not in males. The observed gender differences may be attributed to mortality advantage and son preference in China.

## 1. Introduction

Dyslipidemia is inversely associated with an elevated risk of developing coronary heart disease (CVD), atherosclerosis, ischemic stroke, and acute myocardial infarction.^[1,2]^ In 2002, a nationwide survey conducted in China revealed that the prevalence of dyslipidemia in adults over the age of 18 was 18.6%,^[3]^ which was comparatively lower than the prevalence (53%) observed in the American adult population aged over 20 between 2003 and 2006.^[4]^ The Developmental Origins of Health and Disease^[5]^ thesis proposes that malnutrition during early life increases the risk of chronic disease in adulthood. Previous research has found that prenatal exposure to the Dutch famine increases the risk of CVD,^[6]^ metabolic syndrome,^[7]^ hypertension,^[8]^ diabetes^[9]^ in adulthood. As opposed to the Dutch famine, the Chinese famine, which took place from 1959 to 1962 and resulted in approximately 20 to 30 million deaths,^[10]^ affected the Chinese mainland in the most severe and largest way. Recent studies suggest that exposure to the Chinese famine in early life increases the risk of developing cardiovascular diseases,^[11]^ hyperuricemia,^[12]^ and diabetes.^[13]^ Nonetheless, evidence regarding the association between famine exposure and dyslipidemia is limited. In light of these findings, in this study, dyslipidemia is hypothesized to be associated with famine exposure in later life.

It has been reported that certain factors are positively associated with dyslipidemia, including age, gender, obesity, smoking, and alcohol consumption.^[14–16]^ There has been a growing interest in examining the correlation between famine exposure and dyslipidemia in recent years. Findings from the Dutch famine^[17]^ study demonstrated that individuals who were exposed to famine during early gestation exhibited higher blood lipid levels compared to those who were not exposed. In contrast, the Leningrad famine^[18]^ study did not observe a significant difference in lipid concentration between the exposed and non-exposed groups. Similarly, research on the Chinese famine has yielded inconsistent results regarding the association between early-life famine exposure and dyslipidemia.^[19–21]^

Currently, there is a lack of studies exploring the association between exposure to famine and dyslipidemia during fetal/childhood/adolescence. Furthermore, there is a dearth of research examining gender differences in the association between famine exposure and dyslipidemia. Understanding these factors that contribute to dyslipidemia in adulthood would help to reduce the prevalence of such public health issues. Therefore, our study aimed to analyze the impact of exposure to Chinese famine during fetal/childhood/adolescence and the possible effects on dyslipidemia in adulthood. Our hypothesis posits that famine exposure is associated with dyslipidemia in later life, with gender differences potentially playing a role.

## 2. Methods

### 2.1. Study design

All data were collected from the baseline survey of The China Patient-centered Evaluative Assessment of Cardiac Events (China PEACE) Million Persons Project in Rongchang, spanning from August 2018 to 2022 December. Detailed information about the China PEACE Project^[22]^ has been described previously. Briefly in Ronchang, the population-based survey was conducted in Changyuan, Changzhou, Guangshun, Anfu streets by a 3-stage stratified random sampling method. This survey was complied with the Ethical Committee of National Center for Cardiovascular Disease (NO.2014-574), and informed consent was obtained from all participants prior to their inclusion in the study.

### 2.2. Participants

**Figure 1.**
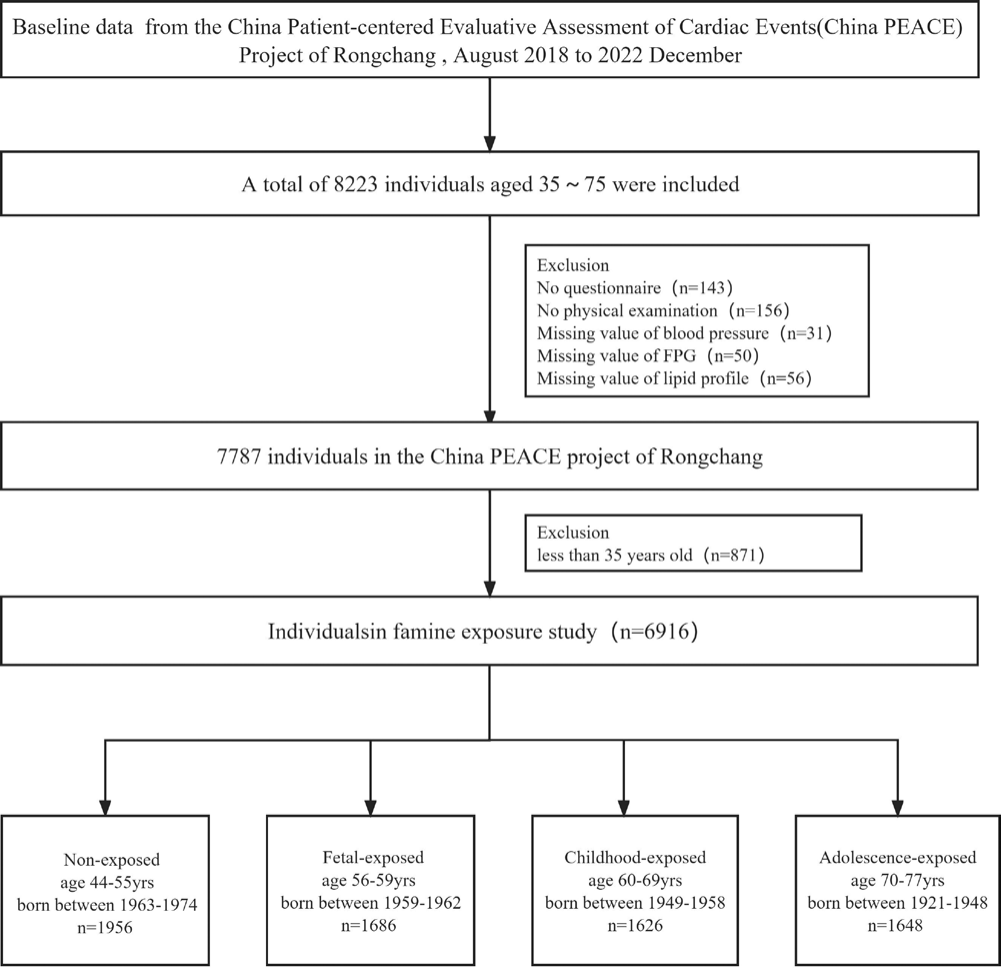
Flowchart on the sample selecting method at each step.

This study was conducted by a 3-stage stratified random sampling method to recruit participants. Firstly, 4 districts were randomly selected in Rongchang. Secondly, approximately 10 to 15 villages were chosen within each selected district. Finally, around 60 to 80 participants were randomly selected. In each selected district, participant allocation was based on the gender and age composition of the Rongchang population. Eligible individuals were recruited based on the following criteria: aged 35 to 75 years old; Han ethnicity; household registered in Rongchang; residing in Rongchang over 6 months; voluntarily agreed to participate in the project, provide biological samples, and cooperate in completing the follow-up; and absence of mental illness, cognitive disorders, or impaired expression abilities. Individuals were excluded based on the following criteria: refusal to cooperate with the entire survey; missing information on questionnaires, physical examination, and blood biochemical tests; and refusal to consent to follow-up. A total of 7787 eligible individuals from the China PEACE project in Rongchang were initially included. However, 871 individuals aged <35 years old were subsequently excluded; 6916 individuals were enrolled into 4 cohorts based on their birthdates: non-exposed, fetal-exposed, childhood-exposed, and adolescent-exposed (Fig. 1). This classification method can be found in the previous study.^[23]^

### 2.3. Variables and measurement

#### 2.3.1. Covariates.

The covariates included in the analysis were gender, age, marital status (married/cohabitated or others), job condition (farmers or others), educational status (primary school or below, junior middle school or high school or above), average yearly income (<20,000 yuan, 20,001–59,999 yuan or ≥60,000 yuan), smoking status (former/current or never), drinking status (former/current or never). The physical activity level (light, moderate, vigorous) was classified into 3 groups according to the Chinese guidelines for data processing and analysis concerning the International Physical Activity Questionnaire.^[24]^ Hypertension was defined as an average systolic blood pressure or diastolic blood pressure ≥ 140/90 mm Hg^[22]^ based on 3 measurements taken in a resting condition or self-reported physician-diagnosed or drug treatment for hypertension. Diabetes was defined as fasting plasma glucose ≥ 7.0 mmol/L according to WHO criteria,^[25]^ or self-reported physician-diagnosed diabetes mellitus, or drug treatment for diabetes. Body mass index (BMI) was calculated by height and weight (BMI = weight/height^2^, kg/m^2^), it was divided into 3 groups (“< 24,” “24–27.9,” and “≥ 28”) according to previous study.^[26]^ Abdominal obesity was defined as a waist circumference of ≥90 cm in males and ≥85 cm in females^[26]^ following established criteria.

#### 2.3.2. Definition of dyslipidemia.

In accordance with the 2016 Chinese guideline for the management of dyslipidemia in adults,^[27]^ individuals were considered to have dyslipidemia if any of these 4 lipid molecules were met: total cholesterol (TC) ≥ 6.22 mmol/L; triglycerides (TG) > 2.30 mmol/L; high-density lipoprotein cholesterol (HDL-C) ≥ 4.10 mmol/L; low-density lipoprotein cholesterol (LDL-C) < 1.00 mmol/L. Self-reported dyslipidemia was also considered in the current study, as determined by a positive response to the following question: “Do you know whether you have been diagnosed with dyslipidemia?”

### 2.4. Statistical analysis

Continuous variables with normal distribution were presented as means ± standard deviation (SD). Median and quartiles were used if the continuous variables did not conform to normal distribution. Demographic characteristics, behavioral factors, and lipid concentrations were compared between exposed and non-exposed cohorts using appropriate statistical tests, including analysis of variance, chi-squared test, Student *t* test, Kruskal–Wallis *H* test, Kruskal–Wallis analysis of variance test, and Bonferroni *χ*^2^-test. Binary logistic regression models were conducted to analyze the association between famine exposure and dyslipidemia while adjusting for confounding factors. Odds ratio (OR) and 95% confidence interval (CI) were calculated. The statistical analysis was performed with IBM SPSS 22.0 statistical software (IBM Corp., Armonk, NY). A value of *P* < .05 was considered statistically significant.

## 3. Results

Basic characteristics of the famine-exposed and non-exposed groups were presented in Table 1. A total of 6916 individuals were included, with an average age of (60.11 ± 9.22) years. Among them, there were 1686 individuals exposed to Chinese famine during fetal period, while 1626 and 1648 individuals were exposed to the famine during childhood and adolescence, respectively. The distribution of age, gender, marital status, education level, job conditions, average yearly income, smoking status, drinking status, physical activity level, hypertension, diabetes, dyslipidemia, abdominal obesity, and BMI demonstrated statistical differences among the 4 cohorts (all *P* < .05) (Table 1).

**Table 1:** Basic characteristics of the study participants according to the Chinese famine exposure, Chongqing, China (N = 6916).

Variables	N	Non-exposed (1963–1974)	Fetal-exposed (1959–1962)	Childhood-exposed (1949–1958)	Adolescence-exposed (1921–1948)	*F*/*χ*^2^	*P*
		(n = 1956)	(n = 1686)	(n = 1626)	(n = 1648)		
Age	6916	48.73 ± 3.39	57.22 ± 1.16	63.92 ± 2.65	72.65 ± 2.19	*F* = 2847.204	<.001
Gender							
Male	3372	1176 (60.12)	800 (47.45)	730 (44.90)	666 (40.41)	*χ*^2^ = 157.918	<.001
Female	3544	780 (39.88)	886 (52.55)	896 (55.10)	982 (59.59)		
Marital status							
Married/cohabitated	5934	1822 (93.15)	1474 (87.43)	1424 (87.58)	1214 (73.67)	*χ*^2^ = 293.788	<.001
Others	982	134 (6.85)	212 (12.57)	202 (12.42)	434 (26.33)		
Education level							
Primary school or below	1890	346 (17.69)	616 (36.54)	472 (29.03)	456 (27.67)	*χ*^2^ = 545.257	<.001
Junior middle school	2366	602 (30.78)	332 (19.69)	636 (39.11)	796 (48.30)		
High school or above	2660	1008 (51.53)	738 (43.77)	518 (31.86)	396 (24.03)		
Job conditions							
Farmers	2756	688 (35.17)	596 (35.35)	706 (43.42)	766 (46.48)	*χ*^2^ = 70.958	<.001
Others	4160	1268 (64.83)	1090 (64.65)	920 (56.58)	882 (53.52)		
Average yearly income							
<20,000 yuan	2550	622 (31.80)	634 (37.60)	650 (39.98)	644 (39.08)	*χ*^2^ = 91.159	<.001
20,001 to 59,999 yuan	2524	766 (39.16)	522 (30.96)	568 (34.93)	668 (40.53)		
≥60,000 yuan	1842	568 (29.04)	530 (31.44)	408 (25.09)	336 (20.39)		
Smoking							
Never	5514	1472 (75.26)	1266 (75.09)	1350 (83.03)	1426 (86.53)	*χ*^2^ = 104.762	<.001
Former/Current	1402	484 (24.74)	420 (24.91)	276 (16.97)	222 (13.47)		
Drinking							
Never	3706	872 (44.58)	858 (50.89)	888 (54.61)	1088 (66.02)	*χ*^2^ = 171.826	<.001
Former/Current	3210	1084 (55.42)	828 (49.11)	738 (45.39)	560 (33.98)		
Physical activity							
Light	1466	204 (10.44)	336 (19.93)	458 (28.27)	446 (27.29)	*χ*^2^ = 754.996	<.001
Moderate	1568	222 (11.36)	308 (18.27)	480 (29.63)	558 (34.15)		
Vigorous	3882	1528 (78.20)	1042 (61.80)	682 (42.10)	630 (38.56)		
Hypertension							
No	3144	1218 (62.27)	812 (48.16)	624 (38.38)	490 (29.73)	*χ*^2^ = 425.194	<.001
Yes	3772	738 (37.73)	874 (51.84)	1002 (61.62)	1158 (70.27)		
Diabetes							
No	5880	1786 (91.31)	1382 (81.97)	1350 (83.03)	1362 (82.65)	*χ*^2^ = 85.434	<.001
Yes	1036	170 (8.69)	304 (18.03)	276 (16.97)	286 (17.35)		
Dyslipidemia							
No	5026	1548 (79.14)	1162 (68.92)	1138 (69.99)	1178 (71.48)	*χ*^2^ = 60.243	<.001
Yes	1890	408 (20.86)	524 (31.08)	488 (30.01)	470 (28.52)		
Abdominal obesity							
No	4540	1470 (75.15)	1156 (68.56)	1014 (62.36)	900 (54.61)	*χ*^2^ = 181.515	<.001
Yes	2376	486 (24.85)	530 (31.44)	612 (37.64)	748 (45.39)		
BMI (kg/m^2^)							
<24	2596	738 (37.73)	648 (38.43)	526 (32.35)	684 (41.50)	*χ*^2^ = 35.586	<.001
24 to 27.9	3108	876 (44.79)	720 (42.70)	808 (49.69)	704 (42.72)		
≥28	1212	342 (17.48)	318 (18.86)	292 (17.96)	260 (15.78)		

Distribution of the 4 exposed cohorts was evaluated by analysis of variance (ANOVA) or *χ*^2^-test.

BMI = body mass index.

The lipid profile measures in different exposed cohorts stratified by gender were presented in Table 2. The overall prevalence of dyslipidemia was 27.33%, with a higher prevalence observed among females (31.32%) compared to males (23.13%) (OR = 1.515, 95% CI: 1.362–1.687). Compared to the non-exposed cohorts, the 3 exposed groups exhibited a statistical difference in females (*P* < .05), but not in males (*P* > .05). Females exposed to famine during the fetal stage showed a significant increase in TC, TG, and LDL-C levels; Childhood-exposed females had significantly higher levels of plasma TC, TG, HDL-C, and LDL-C levels; Adolescence-exposed females had significantly higher levels of plasma TG (Table 2).

**Table 2:** Fasting lipid concentrations, prevalence of dyslipidemia in males and females by life stages when exposure to Chinese famine.

Variables	Non-exposed	Fetal-exposed	Childhood-exposed	Adolescence-exposed
	(1963–1974)	(1959–1962)	(1949–1958)	(1921–1948)
Male				
N	1176	800	730	666
TC level (mmol/L)*	5.05 (4.52–5.53)	5.30 (4.75–5.70)†	5.21 (4.49–5.21)†	5.01 (5.33–5.48)
TG level (mmol/L)*	1.44 (1.00–2.00)	1.36 (0.94–2.04)	1.33 (0.96–1.80)†	1.17 (0.83–1.85)†
HDL-C level (mmol/L)*	1.49 (1.23–1.76)	1.50 (1.18–1.73)	1.37 (1.20–1.75)	1.46 (1.24–1.85)
LDL-C level (mmol/L)*	2.84 (2.31–3.31)	3.07 (2.62–3.63)†	3.01 (2.44–3.71)†	2.69 (2.16–3.12)†
Dyslipidemia (%)	252 (21.43)	200 (25.00)	178 (24.38)	150 (22.52)
Female				
N	780	886	896	982
TC level (mmol/L)*	5.08 (4.51–5.63)	5.24 (4.84–6.17)†	5.44 (4.81–6.13)†	5.12 (4.63–5.81)
TG level (mmol/L)*	1.24 (0.90–1.80)	1.54 (0.96–2.02)†	1.51 (1.08–2.05)†	1.56 (1.08–2.04)†
HDL-C level (mmol/L)*	1.63 (1.36–1.92)	1.63 (1.35–1.88)	1.56 (1.27–1.87)†	1.52 (1.30–1.85)
LDL-C level (mmol/L)*	2.70 (2.28–3.31)	2.95 (2.50–3.58)†	3.05 (2.53–3.59)†	2.81 (2.31–3.47)
Dyslipidemia (%)	156 (20.00)	324 (36.57)†	310 (34.60)†	320 (32.59)†

These continuous variables were non-normality, represented by median (Q1, Q3), and the comparison among groups was performed by Kruskal–Wallis *H* test.

HDL-C = high-density lipoprotein cholesterol, LDL-C = low-density lipoprotein cholesterol, TC = total cholesterol, TG = triglycerides.

* Distribution was significantly different among the 4 birth cohorts (Kruskal–Wallis H test or *χ*^2^-test; *P* < .01).

† Distribution was significantly different between exposed cohort and non-exposed cohort (Kruskal–Wallis analysis of variance test, Bonferroni *χ*^2^-test, *P* < .01).

Table 3 showed logistic regression analysis of risk factors for dyslipidemia. After adjusting for confounding factors, there was no statistically significant difference in the risk of dyslipidemia between males and females (Table 3). Table 4 showed the association between exposure to the Chinese famine and dyslipidemia in different exposed cohorts stratified by gender. After adjusting for confounding factors, the fetal-exposed females (OR = 1.613, 95% CI: 1.179–2.205), childhood-exposed females (OR = 1.857, 95% CI: 1.384–2.491), adolescence-exposed females (OR = 1.531, 95% CI: 1.137–2.060) (all *P* < .05) showed a higher risk of dyslipidemia compared to the non-exposed cohort. There was no similar association observed in exposed males (Table 4).

**Table 3:** Logistic regression analysis of risk factors for dyslipidemia, Chongqing, China (N = 6916).

Variables	OR_crude_ (95% CI)	*P*	OR_adjusted_ (95% CI)	*P*
Age	1.102 (1.006–1.018)	<.001	0.996 (0.989–1.003)	.304
Gender				
Male	1.000 (Ref.)		1.000 (Ref.)	
Female	1.515 (1.362–1.687)	<.001	1.032 (0.818–1.130)	.632
Marital status				
Married/cohabitated	0.783 (0.590–1.038)	.177	0.972 (0.829–1.140)	.728
Others	1.000 (Ref.)		1.000 (Ref.)	
Education level				
Primary school or below	1.000 (Ref.)		1.000 (Ref.)	
Junior middle school	1.167 (1.017–1.340)	.028	1.217 (1.053–1.405)	.008
High school or above	1.219 (1.066–1.393)	.004	1.472 (1.276–1.699)	<.001
Job conditions				
Farmers	0.932 (0.836–1.039)	.164	0.875 (0.674–1.135)	.443
Others	1.000 (Ref.)		1.000 (Ref.)	
Average yearly income				
<20,000 yuan	1.000 (Ref.)		1.000 (Ref.)	
20,001 to 59,999 yuan	0.714 (0.630–0.808)	<.001	0.692 (0.606–0.790)	<.001
≥60,000 yuan	0.825 (0.722–0.942)	.005	0.791 (0.684–0.916)	.002
Smoking				
Never	1.000 (Ref.)		1.000 (Ref.)	
Former/Current	1.154 (0.822–1.619)	.238	1.317 (1.112–1.561)	.001
Drinking				
Never	1.000 (Ref.)		1.000 (Ref.)	
Former/Current	0.903 (0.681–1.198)	.166	0.957 (0.815–1.125)	.597
Physical activity				
Light	1.000 (Ref.)		1.000 (Ref.)	
Moderate	0.970 (0.832–1.132)	.699	0.961 (0.818–1.130)	.633
Vigorous	0.686 (0.601–0.784)	<.001	0.721 (0.623–0.834)	<.001
Hypertension				
No	1.000 (Ref.)		1.000 (Ref.)	
Yes	1.793 (1.607–2.000)	<.001	1.670 (1.483–1.881)	<.001
Diabetes				
No	1.000 (Ref.)		1.000 (Ref.)	
Yes	2.379 (2.076–2.727)	<.001	2.093 (1.814–2.414)	<.001
Abdominal obesity				
No	1.000 (Ref.)		1.000 (Ref.)	
Yes	1.304 (1.169–1.456)	<.001	0.953 (0.839–1.082)	.455
BMI (kg/m^2^)				
<24	1.000 (Ref.)		1.000 (Ref.)	
24–27.9	1.767 (1.564–1.995)	<.001	1.726 (1.516–1.966)	<.001
≥28	1.788 (1.533–2.086)	<.001	1.511 (1.268–1.800)	<.001

Evaluating the association between different factors and dyslipidemia by the binary logistic regression model.

OR_crued_: unadjusted.

OR_adjusted_: adjusted for age, marital status, job conditions, educational status, average yearly income, smoking and drinking status, physical activity, hypertension, diabetes, abdominal obesity, and body mass index.

BMI = body mass index, CI = confidence interval, OR = odds ratio, Ref. = defined as reference.

**Table 4:** Association of exposure to the Chinese famine with dyslipidemia.

Variables	Non-exposed (1963–1974)	Fetal-exposed (1959–1962)	Childhood-exposed (1949–1958)	Adolescence-exposed (1921–1948)
Male				
Model 1	Ref.	1.222 (0.989–1.511)	1.182 (0.950–1.472)	1.066 (0.848–1.340)
Model 2	Ref.	1.397 (0.983–1.884)	0.900 (0.684–1.184)	0.894 (0.669–1.194)
Female				
Model 1	Ref.	2.306 (1.846–2.880)	2.116 (1.693–2.645)	1.934 (1.551–2.410)
Model 2	Ref.	1.613 (1.179–2.205)*	1.857 (1.384–2.491)*	1.531 (1.137–2.060)*

Evaluating the gender risk of 3 exposed cohorts with non-exposed as a reference by the binary logistic regression model

Model 1: unadjusted.

Model 2: adjusted for age, marital status, job conditions, educational status, average yearly income, smoking and drinking status, physical activity, hypertension, diabetes, abdominal obesity, and body mass index.

Ref. = defined as reference.

* *P* < .01.

## 4. Discussion

We observed that exposure to Chinese famine during fetal/childhood/adolescence periods was associated with an increased risk of dyslipidemia in female adults. However, this association was not observed in male adults. Our study contributes new evidence to the hypothesis of Developmental Origins of Health and Disease, suggesting that the detrimental consequences of undernutrition resulting from exposure to famine can extend beyond the “first 1000 days.”

Studies have investigated the relationship between dyslipidemia and famine exposure. This study adds new and further evidence to previous studies. We found that the prevalence of dyslipidemia for female adults exposed to malnutrition in fetal stage was significantly higher compared to the non-exposed cohort, which was consistent with the previous studies in China.^[28–30]^ Hu^[31]^ et al also demonstrated the coexistence of famine exposure in early life and adult obesity is associated with a higher risk of developing dyslipidemia in later life. However, these studies primarily focused on early life exposure to famine. Another study^[21]^ based on the China Health and Nutrition Survey conducted in 2009 found that there was a higher prevalence of dyslipidemia in fetal, childhood, and adolescence-exposed groups compared to the unexposed group. However, the gender differences were not investigated in this study. Two other studies from European Jews^[32,33]^ exposed to the Jewish Holocaust during 1940 to 1945 found that starvation in early infancy was positively correlated with dyslipidemia. A Korean study^[34]^ revealed that females who were exposed to the Korean War during the prenatal period and early childhood exhibited higher triglyceride levels. However, such positive association was not obtained in the Dutch Hunger Winter Families Study,^[35]^ which found that prenatal malnutrition in women had an elevated level of TC and triglyceride concentrations, but the association with dyslipidemia was not observed. Similarly, the Leningrad Siege^[18]^ study found no difference between the individuals exposed to starvation in utero and those starved during infancy. Inconsistent findings can be attributed to the duration and severity of the famine, ethnic differences, and China’s rapid economic development in recent years.

We observed that childhood/adolescence-exposed to Chinese famine resulted in decreased levels of TG in males, which is consistent with Wang’s^[19]^ study. Furthermore, fetal and childhood exposure to famine was associated with higher levels of TC and LDL-C in females. However, we did not find a consistent association with HDL-C across the 3 exposed cohorts. Zheng^[20]^ et al found that there were no statistically significant differences between fetal and postnatal famine exposure groups in terms of TC, TG, HDL-C, and LDL-C compared to the non-exposed, which was aligned with Stanner’s^[18]^ study, which showed that fetal and childhood exposure to famine increased LDL-C, TC, TG levels in adulthood, similar to the previous studies.^[20,35]^ Interestingly, our study revealed that childhood and adolescent females exposed to famine had decreased levels of HDL-C. In contrast, Wang^[19]^ et al indicated the adolescence/adult-exposed group had lower levels of HDL-C compared to the non-exposed and Zheng^[20]^ et al found that HDL-C levels in fetal and early childhood exposure group were higher than the non-exposed group in females, which is inconsistent with our findings. We speculate that these discrepancies may be attributed to differences in the age of the participants enrolled, ethnic differences, variations in severe food shortages in different regions, and the influence of hypertension and diabetes on blood lipid concentration.

Why gender difference was observed in our study might be linked with mortality advantage and son preference in China. Firstly, Chinese traditional culture exhibits a preference for sons.^[36]^ Parents were more likely to protect boys from tough circumstances, boys might have the priority of getting enough food or nutrition resources, while girls might be abandoned or sold to wealthier families for money to create more opportunities for boys. Consequently, women who survived during famine periods may still have experienced prolonged malnutrition, potentially worsening their health outcomes. Secondly, during famines, males generally exhibit a mortality advantage compared to females, particularly during fetus and fetal phase.^[36]^ Male fetuses are more susceptible to adverse environmental conditions, which lead to a lower survival rate. The reason may be due to the fact that male fetuses grow faster in the womb and require a larger share of placenta resources at the expense of their own nutrition. This increased vulnerability to malnutrition may lead to higher mortality rates among male fetuses. As a result, the males who survive are likely to be relatively healthier compared to those who died prematurely.^[37]^ Further research is necessary to fully understand the mechanisms underlying these observations and to explore additional contributing factors.

Our study has several strengths. Firstly, to our knowledge, we were the first to examine gender differences in the association between famine exposure and dyslipidemia within local general population. This unique focus contributes to the existing studies by shedding light on a previously understudied aspect of the relationship. Secondly, the same trained staff conducted data collection in each selected district, ensuring rigorous quality and minimizing potential variations in data acquisition. Thirdly, our study population was general population living in Rongchang. This distinction is crucial as it differs from hospital patients or individuals undergoing medical examinations, thus making our findings more representative and externally generalizable. Despite these strengths, several limitations also should be mentioned. Firstly, due to the absence of clear beginning and end dates for the Chinese Great Famine, we relied on birthdate of participants to define famine exposure cohorts, this made it impossible to categorize participants accurately based on their date of exposure. Secondly, our study found positive associations between famine exposure and dyslipidemia, but we were unable to conclude which specific period was the most critical and sensitive in terms of its impact on dyslipidemia. Based on China’s recent history, the famine in China might have begun in 1959, but it may have extended until 1979, which marked the start of reform and opening up in China. Consequently, famine exposure may have a more complex and long-term impact on health outcomes. Thirdly, the lack of information about individual energy intake during the famine period also prevented us from accurately attributing the risk of famine exposure and dyslipidemia. Although we adjusted for some lifestyle factors in our study, unmeasured confounding factors may still be present. Lastly, a cross-sectional study with a small sample size limits our ability to establish an association between famine exposure and dyslipidemia. Further longitudinal studies with larger sample sizes are needed to provide more robust evidence in this association.

Our research has the potential to make a meaningful impact on female health. The findings suggest that malnutrition during different life stages can increase the risk of dyslipidemia in females. This underscores the importance of ensuring adequate nutrition throughout the lifespan to prevent dyslipidemia in adulthood. Furthermore, our study highlights the need for government intervention and policy measures to address the nutritional needs of females during various stages of growth and development. By implementing appropriate interventions, such as promoting healthy eating habits and providing access to nutritious food, the incidence of chronic diseases, including dyslipidemia, can be effectively reduced.

## 5. Conclusions

In summary, based on the Chinese Great Famine that occurred from 1959 to 1962, we conducted a thorough assessment of the relationship between famine exposure and the risk of dyslipidemia. We employed a rigorous research design and utilized robust analysis methods to effectively control for confounding factors. The findings reveal that females exposed to the Chinese famine during fetal/childhood/adolescence increase the risk of dyslipidemia in adulthood. Additionally, relying on dieting as a method to achieve weight loss goals may further amplify the risk of dyslipidemia in later life.

## Acknowledgments

We are deeply honored to have participated in The China Patient-centered Evaluative Assessment of Cardiac Events (China PEACE) Million Persons Project. We express our utmost gratitude to all the participants. Furthermore, we extend our sincere appreciation for the invaluable support from our team members in the completion of this research endeavor. We would also like to acknowledge the language polishing service provided by Home for Researchers (www.home-for-researchers.com), which greatly enhanced the quality of our manuscript.

## Author contributions

**Conceptualization:** Huali Xiong, Daiqiang Liu.

**Data curation:** Huali Xiong, Daiqiang Liu, Dayi Tang, Fengxun Ma.

**Formal analysis:** Huali Xiong, Daiqiang Liu.

**Funding acquisition:** Huali Xiong, Daiqiang Liu.

**Investigation:** Huali Xiong, Daiqiang Liu, Dayi Tang.

**Resources:** Fengxun Ma.

**Software:** Dayi Tang, Fengxun Ma.

**Validation:** Fengxun Ma.

**Writing – original draft:** Huali Xiong, Daiqiang Liu.

**Writing – review & editing:** Huali Xiong, Daiqiang Liu.
